# Angiotensin-converting enzyme insertion/deletion polymorphism, 24-h blood pressure profile and left ventricular hypertrophy in hypertensive individuals: a cross-sectional study

**DOI:** 10.1186/s40001-015-0166-9

**Published:** 2015-09-04

**Authors:** Luciana Neves Cosenso-Martin, Renan Oliveira Vaz-de-Melo, Luana Rocco Pereira, Cláudia Bernardi Cesarino, Juan Carlos Yugar-Toledo, José Paulo Cipullo, Marcela Augusta de Souza Pinhel, Dorotéia Rossi Silva Souza, José Fernando Vilela-Martin

**Affiliations:** Hypertension Clinic, Internal Medicine Department, State Medical School in São José do Rio Preto (FAMERP) and Hospital de Base, Ave Brig. Faria Lima 5416, São José do Rio Preto, São Paulo, 15090-000 Brazil; Molecular Biology Department, FAMERP, São Paulo, Brazil

**Keywords:** Angiotensin-converting enzyme, Hypertension, Left ventricular hypertrophy, Nocturnal dipping, Polymorphism, Target organ damage

## Abstract

**Background:**

The absence of nocturnal blood pressure dipping (ND) identified by 24-h ambulatory blood pressure monitoring (ABPM) correlates with a worse cardiovascular prognosis. The renin–angiotensin system influences blood pressure levels and the occurrence of target organ damage (TOD). Thus, the aim of this study was to correlate the angiotensin-converting enzyme gene (ACE) insertion/deletion **(**I/D) polymorphism with the 24-h blood pressure profile and TOD in hypertensive individuals.

**Methods:**

155 non-diabetic hypertensive individuals on antihypertensive treatment underwent ABPM. Peripheral blood samples were drawn for biochemistry and genetic analysis of the ACE I/D polymorphism by polymerase chain reaction. ND was defined as ≥10 % differences in the mean systolic blood pressure (BP) during wakefulness and sleep.

**Results:**

There were no differences in clinical or biochemical variables or TOD in respect to ND status, except for higher BP levels during sleep (*p* < 0.001) in non-dippers. There was significant difference in the prevalence of left ventricular hypertrophy (LVH) between ACE genotypes (II: 13.0 %; ID: 34.1 %; DD: 46.5 %; *p* value = 0.024) with an increased risk in carriers of the DD genotype (OR = 5.80; IC 95 % 1.50–22.44; *p* value = 0.011). Carriers of the D allele had higher systolic BP during wakefulness and by ABPM (*p* < 0.05), higher left ventricular mass (117.3 ± 50.0 vs. 100.3 ± 25.7; *p* value = 0.017) and higher prevalence of LVH (37.4 vs. 12.5 %; OR = 4.14; 95 % IC: 1.17–14.65; *p* value = 0.028), compared to the II genotype.

**Conclusions:**

The DD genotype is associated with a higher prevalence of LVH. The presence of the D allele appears to be associated with higher mean 24-h and wake systolic BP measured by ABPM in hypertensive patients under antihypertensive treatment.

## Background

Hypertension (HT) is a highly prevalent disease associated with increased risk of target organ damage (TOD), including myocardial infarction, stroke and left ventricular hypertrophy (LVH). Changes in blood pressure are influenced by daily activities and interactions with neurohumoral, behavioral and environmental factors. Under physiological conditions, there is a drop in the blood pressure (BP) between wakefulness and sleep, a phenomenon known as nocturnal blood pressure dipping (ND). The absence of ND is associated with TOD and higher mortality [1–4].

Due to the multifactorial etiology of HT, many researchers are studying the association between genetic polymorphisms and HT and its complications. This research studied the angiotensin-converting enzyme (ACE) gene insertion/deletion (I/D) polymorphism, which is characterized by the presence (insertion) or absence (deletion) of an Alu repetitive sequence of 287 base pairs (bp) in intron 16 of the ACE gene [5]. The variability in plasma ACE levels is directly related to the I/D polymorphism; the I allele is associated with a reduced level of this enzyme and lower concentrations of angiotensin II in tissues [5], a condition that apparently protects against TOD.

The ACE I/D polymorphism is linked to risk for myocardial infarction and cardiovascular events [6–9], LVH [10–12], microalbuminuria [11, 13, 14], thoracic aortic aneurysm in patients with bicuspid or tricuspid aortic valves [15], pregnancy hypertensive disorders [16, 17], and hypertensive emergency [18]. Moreover, this polymorphism is related to inter-individual differences in BP response, for example, a low dose of the hydrochlorothiazide diuretic produced greater BP reductions in men with the DD genotype and women with the II genotype [19]. Therefore, this polymorphism may also have a role in the genesis of TOD, which is commonly found in hypertensive individuals. Recently, a meta-analysis of 44 articles involving 12,616 subjects, demonstrated the contribution of the I/D polymorphism to susceptibility for LVH [20].

The findings reported in the literature in respect to the relationship of this polymorphism with BP levels and TOD are controversial [14, 21–31]. Thus, this study aimed to characterize the anthropometric, biochemical, and echocardiographic data and blood pressure profile of non-diabetic hypertensive individuals and to investigate the influence of the ACE I/D polymorphism on 24-h BP levels and on TOD.

## Methods

This project was approved by the Research Ethics Committee of the State Medical School in São José do Rio Preto (no. 169/2008). All participants were informed about the purpose of the work and gave their written informed consent before participating in the research. This was a cross-sectional study, which enrolled 155 hypertensive individuals followed-up at a university hospital outpatient clinic. The exclusion criteria were age under 40 years, history of valvular disease, systolic/diastolic dysfunction, diabetes mellitus (DM), body mass index (BMI) ≥35 kg/m^2^, creatinine clearance <30 mL/min/m^2^, and secondary hypertension.

An investigative protocol was used to collect information on age, gender, medicines taken, and comorbidities. Data regarding DM, strokes, MI, and dyslipidemia (DLP) were used to assess comorbidities. Patients who had presented with two or more fasting glycemia levels ≥126 mg/dL or altered oral glucose tolerance test (≥200 mg/dL) were excluded [32]. History of strokes was based on clinical history, medical records, and the presence of sequels. The diagnosis of MI was based on clinical history and confirmed by an analysis of medical records that described enzymatic alterations (troponin and CK-MB), and electrocardiographic alterations suggestive of coronary ischemia and treatment. DLP was identified by total cholesterol (TC), high-density lipoprotein cholesterol fraction (HDL-c) and triglycerides (TG) levels after 12 h of fasting [33]. The following reference values were adopted: TC <200 mg/dL, HDL-c >40 mg/dL, low-density lipoprotein cholesterol fraction (LDL-c) <130 mg/dL and TG <150 mg/dL. The LDL-c fraction was calculated using the formula: LDL-c = TC − HDL-c − TG/5 (for TG <400 mg/dL) [34]. DLP was defined for individuals with alterations in the aforementioned parameters and those who were under treatment with HMG-CoA reductase inhibitors or other hypolipemic drugs.

Weight and height were measured and BMI was calculated using the formula: BMI = weight (kg)/height (m)^2^. Besides stroke and MI, TOD was also considered as the presence of microalbuminuria and left ventricular hypertrophy (LVH). Microalbuminuria was characterized as the average of two 24-h urinary albumin excretion rates ≥20 μg/min.

### Echocardiographic analysis

All echocardiographic examinations were performed by the same experienced echocardiographer. M-Mode echocardiograms were performed under cross-sectional control, with the patient in partial left decubitus position, using an Philips HD15 PureWave ultrasound system and S5-2 Broadband Sector Array Transducer (Andover, MA 01810-1099 -USA) with 2.5- to 3.5-MHz mechanical transducers. Left ventricular (LV) dimensions and mass were assessed from 2D-guided M-mode tracings according to American Society of Echocardiography (ASE) recommendations. M-mode measurements were averaged from three cycles. LV end-systolic, end-diastolic, and stroke volumes were calculated. Ejection fraction was calculated from derived volumes with computed based on the “cubed” or “Teichholtz” equations, but only in the absence of asynergy. 2-D method of Simpson calculated was performed in distorted ventricles [35]. To evaluate the segmental LV function, the ASE recommends adopt 17-segment model scored as 1: normal, 2: hypokinetic, 3: akinetic, 4: dyskinetic, and 5: aneurysmal (diastolically deformed). The ASE also recommends that wall motion be judged by segmental thickening in addition to endocardial motion.

The ASE-recommended formula for estimation of LV mass from LV linear dimensions was performed to define LVH. Left ventricular hypertrophy was defined as left ventricular mass index (LVMI) >115 g/m^2^ in men and >95 g/m^2^ in women [35]. Relative wall thickness was calculated as twice the posterior wall in diastole divided by internal diastolic diameter and was used to estimate the LV geometry concentric (RWT ≥0.42) or eccentric (RWT ≤0.42) hypertrophy [36]. The diastolic dysfunction was evaluated by the ASE and the European Association of Echocardiography (EAE) recommendations. They apply an algorithm that takes into account not only the E/A ratio, but the deceleration time of the E wave, the early and late velocities of the mitral annulus measured by tissue Doppler (e′ and a′), the left atrial volume, the pattern of pulmonary vein flow, and the duration of reversed flow into the pulmonary veins during atrial contraction. The algorithm includes the parameter E/e and also investigates the color M-mode velocity of propagation of the mitral inflow toward the apex. A rapid velocity coincides with normal diastolic function, whereas a slower velocity indicates delayed relaxation [37].

### Ambulatory blood pressure monitoring

BP levels were obtained by ambulatory blood pressure monitoring (ABPM) taking into account the average BP over a 24-h period and differences in the mean awake and sleep BP (presence or absence of ND). ABPM was performed using the Spacelabs 90207 equipment. The equipment was fitted during the daytime and the patient remained with the unit for a period of 24 h. The monitoring process was carried out without patients changing their normal daily activities. The device was programmed to record BP levels at 15-min intervals during daytime, and 20-min intervals at night. All participants were instructed to make notes on their daily activities including meal times, bedtime, and the timing of medications and symptoms. Thus, periods of wakefulness and sleep were defined based on the time recorded by patients in their diaries. ND was defined as ≥10 % drop in systolic BP from wakefulness to sleep [38]. Individuals were classified as dippers (with ND) or non-dippers (without ND). In addition, patients were classified according to the ACE I/D genotypes. Subsequently, patients were grouped as carriers of the D allele (ID and DD genotypes) and compared to those homozygous for the I allele.

### Genetic analysis

Peripheral blood was drawn for the extraction of DNA. The analysis of the genetic variants of the ACE gene was performed by extracting genomic DNA from leukocytes [39], with DNA amplification by conventional polymerase chain reaction (PCR) [40]. The amplification of the polymorphic segment to analyze the ACE I/D polymorphism was performed by initial denaturation with 30 cycles at 94 °C for 1 min, 58 °C for 1 min, 72 °C for 1.5 min, and a final extension of 72 °C for 10 min [40]. Since the amplification of the I allele (490 bp) is less efficient than that of the D allele (190 bp), the specificity of the DD genotyping was increased by amplifying all DD samples using a pair of specific primers for the insertion sequence, with the presence of a DNA fragment of 335 bp confirming the ID genotype [40]. In this case, PCR was performed with 35 cycles at 94 °C for 30 s, 69 °C for 45 s, 72 °C for 2 min, and a final extension of 72 °C for 7 min. The amplification products were subjected to electrophoresis on 1 and 1.8 % agarose gel for samples amplified by the first reaction, and the second reaction, respectively, followed by staining using ethidium bromide and visualization under ultraviolet light.

### Statistical analysis

Descriptive analysis was conducted for the quantitative variables with the presentation of mean values and standard deviations. The Student *t* test and ANOVA were used to analyze quantitative variables and the *χ*^2^ test for qualitative variables. The allelic and genotypic frequencies were compared between the groups using the *χ*^2^ test. The influence of alleles and genotypes on 24-h BP and TOD was detected by calculating cross-products [Odds ratio (OR)] with a 95 % confidence interval (95 % CI). All statistical analyses were performed using the Minitab 15.0 computer program. An alpha error greater than 5 % (*p* value <0.05) was considered statistically significant.

## Results

The anthropometric, biochemical, and echocardiographic data and blood pressure profile of the sample are presented in Table 1. The mean time of HT (16.0 ± 10.1 years), BP levels above the recommended range, and increased values of BMI (28.8 ± 4.9 kg/m^2^) and microalbuminuria (58.1 %) stand out in this sample. On comparing individuals with and without ND, there were no differences between clinical, and biochemical variables or TOD except for lower BP levels during sleep (*p* < 0.001) in patients with ND (Table 2). There was not systolic and diastolic ventricular dysfunction and neither significant areas of infarction scars in the evaluated sample.

**Table 1:** Anthropometric, biochemical, and echocardiographic variables, blood pressure profile and drugs used by hypertensive patients

Variable	Sample (*n* = 155)
Age (years)	63.2 ± 11.4
Gender (male/female)	66/89
Duration of hypertension (years)	16.0 ± 10.1
BMI (kg/m^2^)	28.8 ± 4.9
Smoker, *n* (%)	16 (10.3)
History of stroke, *n* (%)	35 (22.6)
History of myocardial infarction, *n* (%)	7 (4.5)
LVH, *n* (%)	52 (33.5)
Microalbuminuria, *n* (%)	90 (58.1)
Drugs	
Hypolipemiant, *n* (%)	71 (45.8)
Aspirin, *n* (%)	59 (38.1)
Diuretic, *n* (%)	129 (83.2)
ACE inhibitor, *n* (%)	103 (66.5)
Angiotensin II receptor antagonist, *n* (%)	22 (14.2)
Calcium channel blocker, *n* (%)	65 (41.9)
Beta-blocker, *n* (%)	46 (29.7)
Blood pressure levels	
24-h SBP (mmHg)	135.2 ± 17.4
24-h DBP (mmHg)	81.7 ± 11.0
Daytime SBP (mmHg)	137.4 ± 17.6
Daytime DBP (mmHg)	83.7 ± 11.8
Nighttime SBP (mmHg)	129.0 ± 19.2
Nighttime DBP (mmHg)	75.2 ± 11.8
SBP dipping (%)	5.8 ± 8.3
SBP dipping (%)	10.0 ± 9.1
Echocardiographic profile	
Septal thickness (mm)	10.1 ± 1.7
Wall thickness (mm)	9.8 ± 2.9
LV mass (g)	203.2 ± 83.4
LV mass index (g/m^2^)	114.7 ± 47.4
Biochemical profile	
Fasting glycemia (mg/dL)	96.6 ± 15.6
HDL-cholesterol (mg/dL)	56.8 ± 16.1
LDL-cholesterol (mg/dL)	116.2 ± 88.2
Total cholesterol (mg/dL)	187.3 ± 35.8
Triglycerides (mg/dL)	133.5 ± 78.2
Creatinine (mg/dL)	1.5 ± 1.7
Microalbuminuria (µg/min)	33.6 ± 46.3

*BMI* body mass index, *LVH* left ventricular hypertrophy, *ACE* angiotensin-converting enzyme, *DBP* diastolic blood pressure, *SBP* systolic blood pressure, *LV* left ventricle

**Table 2:** Studied variables and genotype distribution according to the nocturnal dipping

Variable	Dippers (*n* = 50)	Non-dippers (*n* = 105)	*p* value
Age (years)	62.8 ± 12.0	63.5 ± 11.1	NS
Gender (Male/Female)	22/28	44/61	NS
Duration of hypertension (years)	14.7 ± 11.1	16.6 ± 9.6	NS
BMI (kg/m^2^)	28.3 ± 4.8	29.0 ± 5.0	NS
Smoker, *n* (%)	6 (12.0)	10 (9.5)	NS
History of stroke, *n* (%)	12 (24.0)	23 (21.9)	NS
History of myocardial infarction, *n* (%)	1 (2.0)	6 (5.7)	NS
LVH, *n* (%)	16 (34.0)	36 (34.6)	NS
Microalbuminuria, *n* (%)	26 (52.0)	64 (60.9)	NS
Drugs			
Hypolipemiant, *n* (%)	18 (36.0)	53 (50.5)	NS
Aspirin, *n* (%)	15 (30.0)	44 (41.9)	NS
Diuretic, *n* (%)	44 (88.0)	85 (80.9)	NS
ACE inhibitor, *n* (%)	31 (62.0)	72 (68.6)	NS
Angiotensin II receptor antagonist, *n* (%)	7 (14.0)	15 (14.3)	NS
Calcium channel blocker, *n* (%)	24 (48.0)	41 (39.0)	NS
Beta-blocker, *n* (%)	11 (22.0)	35 (33.3)	NS
Blood pressure levels			
24-h SBP (mmHg)	134.6 ± 19.0	135.6 ± 16.6	NS
24-h DBP (mmHg)	81.7 ± 9.9	81.7 ± 11.6	NS
Daytime SBP (mmHg)	140.1 ± 19.6	136.1 ± 16.6	NS
Daytime DBP (mmHg)	85.8 ± 10.4	82.7 ± 12.3	NS
Nighttime SBP (mmHg)	118.7 ± 16.7	133.9 ± 18.4	<0.001
Nighttime DBP (mmHg)	69.3 ± 9.2	78.0 ± 11.9	<0.001
SBP dipping (%)	15.1 ± 4.6	1.4 ± 5.6	<0.001
SBP dipping (%)	19.0 ± 5.7	5.6 ± 7.0	<0.001
Echocardiographic profile			
Septal thickness (mm)	10.1 ± 1.6	10.1 ± 1.8	NS
Wall thickness (mm)	9.8 ± 1.5	9.9 ± 3.4	NS
LV mass (g)	200.0 ± 64.4	204.8 ± 91.3	NS
LV mass index (g/m^2^)	112.8 ± 31.8	115.6 ± 53.1	NS
Biochemical profile			
Fasting glycemia (mg/dL)	95.6 ± 18.9	97.1 ± 13.8	NS
HDL-cholesterol (mg/dL)	56.7 ± 15.2	56.8 ± 16.6	NS
LDL-cholesterol (mg/dL)	110.8 ± 22.7	109.2 ± 31.6	NS
Total cholesterol (mg/dL)	193.6 ± 28.4	184.3 ± 38.6	NS
Triglycerides (mg/dL)	138.8 ± 71.6	130.9 ± 81.4	NS
Creatinine (mg/dL)	1.2 ± 0.3	1.3 ± 1.1	NS
Microalbuminuria (µg/min)	38.2 ± 59.1	31.6 ± 39.7	NS
Genotype			
II, n (%)	8 (16.0)	16 (15.2)	NS
ID, *n* (%)	25 (50.0)	62 (59.0)	
DD, *n* (%)	17 (34.0)	27 (25.7)	
Genotype combination			
II, *n* (%)	8 (16)	16 (15.3)	NS
D_, *n* (%)	42 (84)	89 (84.7)	

*BMI* body mass index, *LVH* left ventricular hypertrophy, *ACE* angiotensin-converting enzyme, *DBP* diastolic blood pressure, *SBP* systolic blood pressure, *LV* left ventricle, *NS* non-significant (*p* > 0.05)

When grouped according to the genotype of the ACE I/D polymorphism, the groups did not significantly differ in respect to mean age, gender, BMI and duration of HT. There were no statistically significant differences between the three genotypes (II, ID, and DD) and mean 24-h BP by ABPM (Table 3). However, there was a significant difference in the prevalence of LVH between the ACE genotypes (II: 13.0 %; ID: 34.1 %; DD: 46.5 %; *p* value = 0.024). By logistic regression (Table 4), carriers of the DD genotype had higher risk of LVH compared to those with the II genotype (OR: 5.8; 95 % CI 1.50–22.44; *p* value = 0.011). There was no significant difference between genotypes in respect to the other TOD investigated in this study.

**Table 3:** Distribution of studied variables according to the genotypes of the ACE I/D polymorphism

Variables	II (*n* = 24)^a^	ID (*n* = 87)^b^	DD (*n* = 44)^c^	*p* value^a × b × c^	DD + ID (*n* = 131)^d^	*p* value^a × d^
Age (years)	61.7 ± 11.9	63.1 ± 10.6	64.3 ± 12.6	NS	63.5 ± 11.3	NS
Gender (male/female)	8/16	41/46	17/27	NS	58/73	NS
Duration of hypertension (years)	16.9 ± 9.9	15.9 ± 10.0	15.7 ± 10.6	NS	15.8 ± 10.2	NS
BMI (kg/m^2^)	27.8 ± 4.0	29.1 ± 4.7	28.8 ± 5.7	NS	29.0 ± 5.1	NS
History of stroke, *n* (%)	9 (37.5)	17 (19.5)	9 (20.5)	NS	26 (19.8)	NS
History of myocardial infarction, *n* (%)	0 (0.0)	5 (5.8)	2 (4.5)	NS	7 (5.3)	NS
LVH, *n* (%)	3 (13.0)	29 (34.1)	20 (46.5)	*0.024*	49 (37.4)	*0.019*
Microalbuminuria, *n* (%)	14 (58.3)	54 (62.0)	22 (50.0)	NS	76 (58.0)	NS
Albuminuria (µg/min)	30.9 ± 29.8	27.7 ± 24.9	46.3 ± 75.0	NS	34.1 ± 48.9	NS
Drugs						
Hypolipemiant,* n* (%)	10 (41.7)	37 (42.5)	24 (54.5)	NS	61 (46.6)	NS
Aspirin, * n* (%)	9 (37.5)	33 (37.9)	17 (38.6)	NS	50 (38.2)	NS
Diuretic, *n* (%)	21 (87.5)	71 (82.6)	37 (84.1)	NS	108 (83.1)	NS
ACE inhibitor, * n* (%)	17 (70.8)	58 (66.7)	28 (63.6)	NS	86 (65.7)	NS
Angiotensin II receptor antagonist, * n* (%)	2 (8.3)	17 (19.5)	3 (6.8)	NS	20 (15.3)	NS
Calcium channel blocker, * n* (%)	10 (41.7)	36 (41.4)	19 (43.2)	NS	55 (42.0)	NS
Beta-blocker, * n* (%)	8 (33.3)	28 (32.2)	10 (22.7)	NS	38 (29.0)	NS
Blood pressure levels						
24-h SBP (mmHg)	129.9 ± 12.6	134.8 ± 16.7	139.1 ± 20.1	NS	136.2 ± 18.0	*0.042*
24-h DBP (mmHg)	79.3 ± 5.9	81.7 ± 10.5	82.9 ± 13.7	NS	82.1 ± 11.7	NS
Daytime SBP (mmHg)	131.8 ± 12.7	136.5 ± 17.1	142.2 ± 19.9	NS	138.4 ± 18.2	*0.036*
Daytime DBP (mmHg)	80.0 ± 9.4	84.0 ± 11.0	85.2 ± 13.9	NS	84.4 ± 12.0	NS
Nighttime SBP (mmHg)	123.8 ± 15.4	129.2 ± 19.0	131.4 ± 21.2	NS	130.0 ± 19.7	NS
Nighttime DBP (mmHg)	73.2 ± 7.0	75.5 ± 11.6	75.6 ± 14.2	NS	75.6 ± 12.5	NS
SBP dipping (%)	5.5 ± 7.9	5.3 ± 8.0	6.9 ± 9.0	NS	5.9 ± 8.4	NS
DBP dipping (%)	8.7 ± 9.0	9.7 ± 9.0	10.9 ± 9.5	NS	10.2 ± 9.2	NS
Echocardiographic profile						
Septal thickness (mm)	9.2 ± 1.1	10.4 ± 1.8	10.0 ± 1.7	*0.007*	10.3 ± 1.8	*<0.001*
Wall thickness (mm)	8.7 ± 1.1	10.2 ± 3.6	9.6 ± 1.6	NS	10.0 ± 3.1	*<0.001*
LV mass (g)	170.1 ± 48.0	213.3 ± 95.1	201.4 ± 68.9	NS	209.3 ± 87.1	*0.003*
LV mass index (g/m^2^)	100.3 ± 25.7	120.1 ± 57.0	111.7 ± 31.7	NS	117.3 ± 50.0	*0.017*

Italic values are significant (*p* < 0.05)

*BMI* body mass index, *LVH* left ventricular hypertrophy, *ACE I/D* angiotensin-converting enzyme insertion/deletion polymorphism, *DBP* diastolic blood pressure, *SBP* systolic blood pressure, *LV* left ventricle, *NS* non-significant (*p* > 0.05)

^a^Information about II genotype

^b^Information about ID genotype

^c^Information about DD genotype

^d^Information about DD + ID genotypes

^a × b × c^Statistical analysis among the groups

^a × d^Statistical analysis between II genotype and sum of ID + DD genotypes

**Table 4:** Logistic regression model for left ventricular hypertrophy according to the genotypes of the ACE I/D

Variable	*β*	Standard error	Odds ratio (95 % CI)	*p* value
LVH (II as reference)			1.00	
ID	1.239	0.660	3.45 (0.95–12.59)	NS
DD	1.757	0.690	5.80 (1.50–22.44)	*0.011*
LVH (II as reference)			1.00	
DD + ID	1.419	0.645	4.14 (1.17–14.65)	*0.028*

Italic values are significant (*p* < 0.05)

*ACE I/D* angiotensin-converting enzyme insertion/deletion polymorphism, *LVH* left ventricular hypertrophy, *NS* non-significant (*p* > 0.05) *p*<0.05 significant

Carriers of the D allele (ID and DD genotypes) had higher awake systolic BP and mean 24-h BP compared to those with the II genotype (*p* value = 0.036 and *p* value = 0.042, respectively) (Table 3) although there were no statistically significant differences between the three genotypes. Moreover, carriers of the D allele also had higher left ventricular mass (209.3 ± 87.1 vs. 170.1 ± 48.0 g; *p* value = 0.003), left ventricular mass index (117.3 ± 50.0 vs. 100.3 ± 25.7 g/m^2^; *p* value = 0.017) and, consequently, higher prevalence of LVH (37.4 vs. 12.5 %; *p* value = 0.019) than individuals with the II genotype. By logistic regression, D allele carriers had a three times higher risk of LVH than those with the II genotype (OR = 4.14; 95 % CI 1.17–14.65; *p* value = 0.028) (Table 4). The allelic and genotypic distributions of the population were in balance according to the Hardy–Weinberg equilibrium (*χ*^2^ = 3.11; *p* > 0.05).

## Discussion

This study demonstrates an association between the D allele of the ACE I/D polymorphism and increased BP, and a correlation of the DD genotype with higher prevalence of LVH in non-diabetic hypertensive patients who despite antihypertensive treatment have poorly controlled BP.

Recently, there has been growing interest in association studies on the I/D polymorphism and ACE plasma levels and, consequently, on angiotensin II concentrations. However, results of studies on this relationship in hypertensive patients are controversial. Thus, while some case–control studies did not confirm any association between the I/D polymorphism and HT [21, 23, 24, 27, 41], others reported a higher frequency of the D allele [28, 31] and DD genotype in subjects with HT [22, 28–30]. In these cases, it is possible that the profile of the sample regarding age, gender and associated diseases may have influenced the distribution of ACE genetic variants. In fact, studies performed in several countries have confirmed this association [22, 28–30]. In a Japanese population, one study found higher systolic BP in subjects aged ≥50 years with the ID genotype compared to II carriers, and higher diastolic BP in individuals with the ID and DD genotypes compared to those with the II genotype [25]. Among the possible explanations for these differences, is that BP increases with age, which may have influenced the polymorphism studied [42].

In the current study, an association was found between the D allele and higher systolic BP during wakefulness and during 24-h ABPM in treated patients, results that are not supported by some other authors [21, 23, 41]. On the other hand, higher BP levels were demonstrated for all evaluated periods (wake, sleep and 24-h) in elderly hypertensive Japanese patients who had the D allele [43]. In addition, Julve et al. [44] reported slightly higher BP levels in patients with the DD genotype, due to higher BP during the nighttime and thus less nocturnal dipping. Although some authors demonstrated this association only in males [22, 45], the authors of a meta-analysis found 79 % greater risk of hypertension in females with the DD compared to those with the II genotype [13].

In the present study, the ACE I/D polymorphism had no influence on ND, which corroborates the results of other authors [21, 23] although one study demonstrated a smaller drop between awake and sleep BP in hypertensive patients with the DD genotype in comparison to carriers of the I allele [44]. In addition, there was no effect of ACE I/D polymorphism or serum ACE levels on circadian variability of blood pressures among pediatric renal transplant patients [46] and in patients with type 1 diabetes [47].

In relation to the association between the ACE I/D polymorphism and TOD in hypertensive individuals, the current research shows that individuals with the DD genotype had an almost fivefold higher risk for LVH compared to those with the II genotype, a condition reported by previous studies [10, 11]. Recently, two meta-analyses demonstrated an association between the ACE I/D polymorphism and LVH [20, 48]. However, other authors did not confirm this association [49, 50]. These conflicting results may reflect the influence of ethnicity, comorbidities, and even of antihypertensive treatment on the ACE I/D polymorphism and TOD associated with HT.

There is evidence that hypertensive patients with the DD genotype present the best antihypertensive response to medications, especially in respect to renin–angiotensin system (RAS) blockers [51, 52]. These studies demonstrated a genetic influence on response to BP treatment, and the effect of RAS blockers on the regression of LVH. Recently, another investigation demonstrated individuals presented with emergency hypertensive (HE) characterized by TOD had a lower frequency of the II genotype and less use of RAS blockers when compared with the hypertensive urgency group, characterized as no TOD. Thus, the risk of an individual having HE was increased in the presence of these two conditions (low frequency of the II genotype and less use of RAS blockers) [18]. On the other hand, one study demonstrated the effect of ramipril in inflammatory response in patients undergoing coronary artery bypass grafts without the ACE I/D gene polymorphism influence [53].

It is known that TOD is more related to the mean 24-h BP levels than office BP levels [1, 2], which highlights the need for ABPM to evaluate hypertensive patients. The absence of ND seems to correlate with TOD in hypertensive individuals [54], although this result was not found in the current study.

In the present study, the DD genotype was associated to higher risk of LVH compared to the II genotype, which suggests that the ACE I/D polymorphism may be more important than ND in the genesis of LVH. In favor of this hypothesis, the results of a meta-analysis found a greater risk for LVH associated with the D allele or DD genotype in untreated hypertensive patients [20]. In addition, DD genotype was also independently associated with worse echocardiographic outcome in patients with non-ischemic heart failure [55]. This is related to an increased neurohormonal response, mainly of the renin–angiotensin aldosterone system [56]. So, it is important to point that LVH is an early TOD associated to cardiac insufficiency. These facts suggest that independent of the treatment, LVH occurs more frequently in individuals with the D allele or DD genotype. Thus, it is important to highlight that it is possible to detect the impact of genetic load on the cardiac phenotype, even with pharmacological treatment.

Some limitations of this study should be mentioned. First, the size of the sample was small, in part, because of the difficulty to select patients due to the exclusion criteria. Second, it was not possible to exclude patients with obstructive sleep apnea, a fact that may influence the frequency of LVH. However, we have excluded patients with BMI ≥35 kg/m^2^, which reduces greatly the possibility of sleep apnea. Finally, the lack of a control group of normotensive individuals, in our viewpoint, was the main limitation of this study. Besides, we can cite two strong points. The study population was separated in two groups, according to nocturnal dipping and the investigation of influence of the ACE I/D polymorphism on 24-h BP levels and on target organ damage.

## Conclusions

To our knowledge, this is the first study that evaluates the ACE I/D polymorphism in respect to ABPM and TOD in Brazilian hypertensive patients. The present study reports two findings: the presence of the D allele of the ACE I/D polymorphism is correlated with higher mean 24-h systolic BP and wake systolic BP, and the DD genotype is associated with higher prevalence of LVH in non-diabetic hypertensive patients on antihypertensive treatment.

## Abbreviations

HT: hypertension
TOD: target organ damage
LV: left ventricular
LVH: left ventricular hypertrophy
BP: blood pressure
ND: nocturnal blood pressure dipping
ACE: angiotensin-converting enzyme
I/D: insertion/deletion
DM: diabetes mellitus
BMI: body mass index
MI: myocardial infarction
DLP: dyslipidemia
TC: total cholesterol
HDL-c: high-density lipoprotein cholesterol
LDL-c: low-density lipoprotein cholesterol fraction
TG: triglycerides
LVMI: left ventricular mass index
ABPM: ambulatory blood pressure monitoring
PCR: polymerase chain reaction
OR: odds ratio
CI: confidence interval
RAS: renin–angiotensin system
